# Correction to “Enhancing the revelation of key genes and interaction networks in non‐small cell lung cancer with major depressive disorder: A bioinformatics analysis”

**DOI:** 10.1002/hsr2.2290

**Published:** 2024-08-30

Gui H, Chen X, Nie Y, Zhang X. Enhancing the revelation of key genes and interaction networks in non‐small cell lung cancer with major depressive disorder: a bioinformatics analysis. *Health Sci Rep*. 2024. 25;7(6):e2167. doi:10.1002/hsr2.2167

In the “ACKNOWLEDGMENTS” section, the text “The Science and Technology Fund project of Guizhou Provincial Health Commission (2023‐3‐5).” was incorrect. This should have read: “The Science and Technology Fund project of Guizhou Provincial Health Commission (gzwkj2024‐084). Qianxinan Prefecture Science and Technology Plan Project (2023‐3‐5).”

We apologize for this error.

